# The role of sentinel node tumor burden in modeling the prognosis of melanoma patients with positive sentinel node biopsy: an Italian melanoma intergroup study (*N* = 2,086)

**DOI:** 10.1186/s12885-022-09705-y

**Published:** 2022-06-03

**Authors:** Saveria Tropea, Paolo Del Fiore, Andrea Maurichi, Roberto Patuzzo, Mario Santinami, Simone Ribero, Pietro Quaglino, Virginia Caliendo, Lorenzo Borgognoni, Serena Sestini, Giuseppe Giudice, Eleonora Nacchiero, Corrado Caracò, Adriana Cordova, Nicola Solari, Dario Piazzalunga, Francesca Tauceri, Paolo Carcoforo, Maurizio Lombardo, Sara Cavallari, Simone Mocellin, Italian Melanoma Intergroup (IMI), Maddalena Cespa, Rosachiara Forcignanò, Gianmichele Moise, Maria Concetta Fargnoli, Caterina Ferreli, Maria Grimaldi, Guido Zannetti, Saverio Cinieri, Giusto Trevisan, Ignazio Stanganelli, Giovanna Moretti, Francesca Bruder, Luca Bianchi, Maria Teresa Fierro, Luigi Mascheroni, Salvatore Asero, Caterina Catricalà, Stefania Staibano, Gaetana Rinaldi, Riccardo Pellicano, Laura Milesi, Marilena Visini, Franco Di Filippo, Leonardo Zichichi, Maria Antonietta Pizzichetta, Carmelo Iacono, Massimo Guidoboni, Giovanni Sanna, Michele Maio, Michele Del Vecchio, Lucia Lospalluti, Leonardi Vita, Annamaria Pollio, Carlo Riberti

**Affiliations:** 1 Soft-Tissue, Peritoneum and Melanoma Surgical Oncology Unit, Veneto Institute of Oncology IOV- IRCCS, 35128 Padua, Italy; 2 Department of Surgery, Oncology and Gastroenterology (DiSCOG), University of Padua, Padua, Italy; 3 Fondazione IRCCS Istituto Nazionale Dei Tumori, Melanoma and Sarcoma Unit, Milan, Italy; 4 Department of Dermatologic Surgery, Città della Salute e della Scienza, Turin, Italy; 5 Department of Medical Sciences, Clinic of Dermatology, University of Turin, Turin, Italy; 6 Dermatologic Surgery Section, Oncologic Department, “Città Della Salute E Della Scienza Di Torino” University Hospital, Turin, Italy; 7 SOC Chirurgia Plastica Ricostruttiva E Melanoma & Skin Cancer Unit, Osp. SM Annunziata, AUSL Toscana Centro, Florence, Italy; 8 U.O.C. Di Chirurgia Plastica Ricostruttiva E Centro Ustioni Policlinico, University of Bari, Bari, Italy; 9 Corrado Caracò M.D., Struttura Complessa Chirurgia Oncologica Melanoma - Istituto Nazionale Tumori-Fondazione “G. Pascale”, Naples, Italy; 10 Department- of Surgical Oncologic and Stomatologic Sciences, University of Palermo, Palermo, Italy; 11 Chirurgia Ospedaliera 1 Ospedale Policlinico San Martino, Genoa, Italy; 12 Chirurgia Generale 1, Ospedale Papa Giovanni XXIII, Bergamo, Italy; 13 Chirurgia E Terapie Oncologiche Avanzate Ospedale “GB.Morgagni-L.Pierantoni” - AUSL Forlì, Forlì, Italy; 14 UOC Chirurgia II Azienda Ospedaliera Universitaria Di Ferrara, Ferrara, Italy; 15 Dermatology Unit, Department of Specialistic Medicine, ASST Dei Sette Laghi, Varese, Italy; 16 M.D. S. C. Chirurgia Generale ASST Carlo Poma, Mantua, Italy; 17 Italian Melanoma Intergroup (IMI), 16121 Genoa, Italy

**Keywords:** Melanoma, Treatment of cutaneous melanoma, Prognostic factors, Tumor burden, Risk stratification, Overall survival, Metastatic melanoma in the sentinel nodes, Completion lymph node dissection, CLND, Nomogram

## Abstract

**Background:**

The management of melanoma patients with metastatic melanoma in the sentinel nodes (SN) is evolving based on the results of trials questioning the impact of completion lymph node dissection (CLND) and demonstrating the efficacy of new adjuvant treatments. In this landscape, new prognostic tools for fine risk stratification are eagerly sought to optimize the therapeutic path of these patients.

**Methods:**

A retrospective cohort of 2,086 patients treated with CLND after a positive SN biopsy in thirteen Italian Melanoma Centers was reviewed. Overall survival (OS) was the outcome of interest; included independent variables were the following: age, gender, primary melanoma site, Breslow thickness, ulceration, sentinel node tumor burden (SNTB), number of positive SN, non-sentinel lymph nodes (NSN) status. Univariate and multivariate survival analyses were performed using the Cox proportional hazard regression model.

**Results:**

The 3-year, 5-year and 10-year OS rates were 79%, 70% and 54%, respectively. At univariate analysis, all variables, except for primary melanoma body site, were found to be statistically significant prognostic factors. Multivariate Cox regression analysis indicated that older age (*P* < 0.0001), male gender (*P* = 0.04), increasing Breslow thickness (*P* < 0.0001), presence of ulceration (*P* = 0.004), SNTB size (*P* < 0.0001) and metastatic NSN (*P* < 0.0001) were independent negative predictors of OS.

**Conclusion:**

The above results were utilized to build a nomogram in order to ease the practical implementation of our prognostic model, which might improve treatment personalization.

## Background

The standard treatment of cutaneous melanoma has been wide excision of the primary tumor combined with sentinel node (SN) biopsy for staging purposes, the SN status being one of the strongest predictors of prognosis [1–3]_._ For many years, completion lymph node dissection (CLND) has been the standard approach for patients with metastatic SN. However, with publication of the Multicenter selective Lymphadenectomy-2 trial (MSLT2) and the German Dermatologic Cooperative Oncology Group study (DeCOG-SLT) and considering the evolving landscape of adjuvant therapy in melanoma patients, immediate CLND is not recommended in the first course [4–8]. MSLT-2 and DeCOG-STLT showed that CLND increases the rate of regional disease control and provides prognostic information but is not associated with an improved melanoma specific survival [5, 6]. However, these trials, the results of which might lead to abandon the practice of CLND, had some limitations. First, retrospective series produced varied results and were subject to a considerable risk of select bias; next, there were differences in clinic-pathologic features of the patient cohorts between centers; moreover, most patients, enrolled in these studies, had a low-volume nodal tumor burden [5, 7, 9–11]. In the end, international guidelines such as those issued by the National Comprehensive Cancer Network, recommend to discuss with the patient the benefit of CLND mainly based on the risk of harboring additional lymph node metastatic disease. In analogy with breast cancer, the current availability of an effective adjuvant therapy (either targeted therapy or immunotherapy) for patients with SN positive melanoma is further pushing against the use of CLND [5, 7, 12, 13]. However, omitting CLND could result in underestimation of patients at high risk progression and so in an impaired selection for adjuvant therapy. In fact, there is an approximately 20% of melanoma patients harboring metastatic disease in non-sentinel nodes [5, 6, 8, 13–16]. In order to predict the presence of disease in NSN for follow up personalization in clinical practice and for patient risk stratification, Italian Melanoma Intergroup (IMI) built a nomogram for prediction NSN status in melanoma patients with positive SNB [4]. On the other hand, patients with minimal residual disease in the SN often experience a favorable clinical outcome: in this subgroup, adjuvant therapy might represent an overtreatment since surgery is likely to have already completely eradicated the tumor [4, 17].

In this challenging landscape, adjuvant therapy decision making would greatly benefit from the identification independent prognostic factors improving the risk stratification, which ultimately would enable physicians to optimize the management of melanoma patients with positive SNB [1–13].

In 2017, the 8^th^ edition of American Joint Committee of Cancer (AJCC) melanoma classification was published and one of the major changes was the criteria to allocate patients in stage III. However, no new prognostic factor was introduced [17, 18].

Tumor burden (TB), the maximum diameter of the largest tumor deposit in the SN, has been advocated as a potentially useful prognostic parameter in stage III patients. Although it is not incorporated in the AJCC staging system, the AJCC panel acknowledged the importance of TB and recommended the assessment of tumor load to be performed by every melanoma Center [17–19]_._

## Methods

### Study design

This is a retrospective study based on information from prospectively maintained databases managed by 13 Italian centers belonging to the Italian Melanoma Intergroup (IMI).

This study aimed to test the hypothesis that TB significantly contributes to predict overall survival (OS) in patients with metastatic melanoma in the sentinel node(s) who underwent CLND. The results were used to build a prognostic nomogram of clinical use.

Data regarding a cohort of 2,086 patients were reviewed. We considered the following covariates: age (as a continuous value), gender (male vs female), melanoma body site (head and neck, trunk, limbs), Breslow thickness (as a continuous value), ulceration (absent vs present), number of positive sentinel nodes (as a continuous value), sentinel node TB (0.01 mm-0.4 mm; 0.41 mm-0.96 mm; 0.97 mm- 3 mm; 3.1 mm-35 mm), non-sentinel lymph node status (positive vs negative).

The main inclusion criteria for SNB was melanoma with Breslow thickness ≥ 1 or melanoma with thinner tumors with adverse prognostic features such as ulceration, a high mitotic rate or Clark level IV o V. The main inclusion criteria for CLND were positive SNB and lack of clinical or radiological evidence of metastatic disease (all patients were M0).

The pathology protocols to assess primary melanoma features, SN and NSN status were shared by all 13 IMI Centers.

T coefficients obtained from the multivariable model were used to set up a nomogram for practical use.

### Statistical analysis

OS curve was estimated using the Kaplan Meier method. Univariate and multivariate survival analyses were performed using the Cox proportional hazard regression model.

In order to check for model overfitting we used the bootstrap method (1,000 replications). Briefly, random samples drawn with replacement from the original data set are created with the same size as the original series; the performance index of the model built on the entire cohort is always better than the average of the indices calculated in each replication. The difference between the two is an estimate of the model overfitting (optimism) and the average value of the indices is considered the unbiased estimate of how well the model would perform in future data set. The alpha level of significance was set at 5%.

All analyses were performed using Stata 11.2 SE software (StataCorp LLc, College Station, TX, USA).

## Results

Patients diagnosed with cutaneous melanoma between 2000 and 2018 were studied.

Patients and tumor characteristics are reported in Table 1. Univariate and multivariate survival analyses are reported in Table 2.

**Table 1:** Patients and tumor characteristic

Variable	Median (range)	n	%
Age (years)	56 (4–90)		
Gender			
Male		1203	58
Female		883	42
Melanoma body site			
Head and neck		130	6
Trunk		1008	48
Limbs		948	46
Breslow (mm)	3,53 (0,30–40)		
Ulceration			
Yes		960	46
Not		1126	54
SN tumor burden	2,59 (0,01–35)		
0,01–0,4 mm		577	27
0,41–0,96 mm		471	23
0,97–3 mm		586	28
3,1–35 mm		451	22
NSN status			
Positive		453	22
Negative		1633	78

**Table 2:** Results of univariate and multivariate analysis

	N	**5-YSR (%)**	**10-YSR (%)**	**UNIVARIATE**	***P*-value**	**MULTIVARIATE**	***P*-value**
				**HR**		**HR**	
**Variable**							
**Age**				1,02 (1,02–1,03)	** < 0,0001**	1,01 (1,01–1,02)	** < 0,0001**
**Gender**							
**Female**	883	74	60	Reference		Reference	
**Male**	1203	63	50	1,33 (1,12–1,58)	**0,0005**	1,19 (1,00–1,41)	**0,04**
**Melanoma body site**					**0,58**		
**Breslow**				1,08 (1,06–1,10)	** < 0,0001**	1,06(1,04–1,08)	** < 0,0001**
**Ulceration**							
**Not**	1126	76	63	Reference		Reference	
**Yes**	960	60	42	1,86 (1,57–2,20)	** < 0,0001**	1,30 (1,08–1,56)	**0,004**
**Number of positive SN**				1,22 (1,06–1,41)	**0,0066**		**0,16**
**SN tumor burden**							
**0,01–0,4 mm**	577	82	68	Reference			
**0,42–0,96 mm**	471	76	58	1,27(1,01–1,59)			
**0,97–3 mm**	586	66	52	1,89 (1,54–2,34)			
**3,1–35 mm**	452	49	34	3,51 (2,74–4,49)	** < 0,0001**	1,04 (1,03–1,06)	** < 0,0001**
**NSN Status**							
**Negative**	1633	75	61	Reference			
**Positive**	453	46	31		** < 0,0001**	2,06(1,72–2,5)	** < 0,0001**

**Fig. 1:**
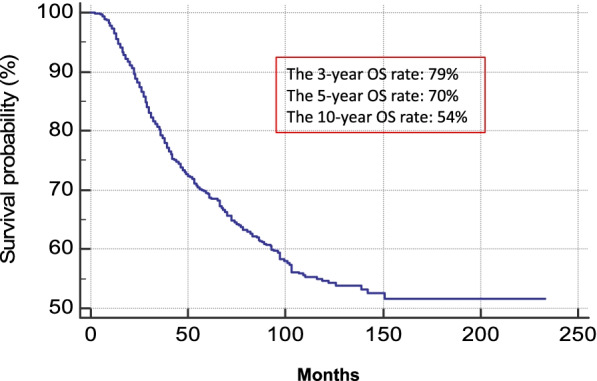
OS Kaplan Meier curve

The 3-year, 5-year and 10-year OS rates were 79%, 70% and 54%, respectively (Fig. 1). At univariate analysis, all variables, except for primary melanoma body site, were found to be statistically significantly associated with patient prognosis. Multivariate Cox regression analysis indicated that age (HR = 1.01; 95% CI: 1.01–1.02, *p* < 0.0001), male sex (HR = 1.19; 95% CI: 1.00–1.41, *p* = 0.0005), Breslow (HR = 1.06; 95% CI: 1.04–1.08, *p* < 0.0001), NSN metastatic status (HR = 2.06; 95% CI: 1.72–2.50, *p* < 0.0001), ulceration (HR = 1.30; 95% CI: 1.08–1.56, *p* < 0.0001) and the diameter of sentinel node metastasis (HR = 1.04; 95% CI: 1.03–1.06, *p* < 0.0001) were independent negative prognostic factors of OS. Number of positive SN did not result a statistically significant predictor of OS.

**Fig. 2:**
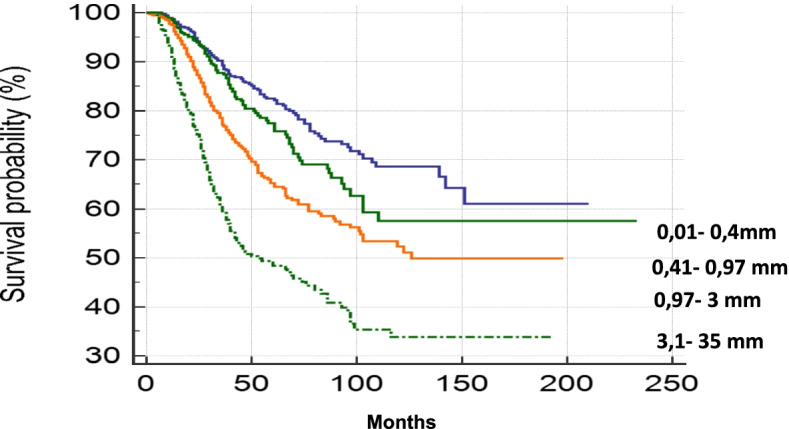
OS according to TB

**Fig. 3:**
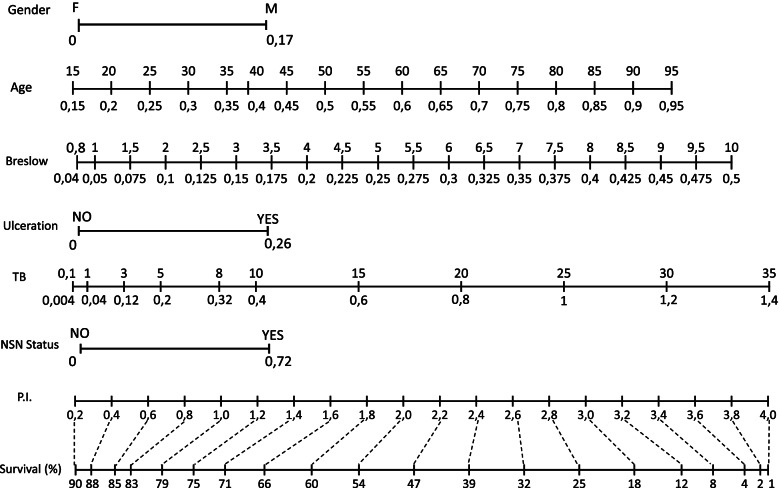
Prognostic nomogram

As regards TB, we grouped SN metastasis diameter in quartiles and so we identified 4 subgroups of patients (Fig. 2). We found that SN TB was an important predictor of OS with a progressive worsening prognosis from first (0.01 mm-0.04 mm) to fourth subgroup (3.1 mm-35 mm). In details, the latest subgroup had a significantly poor prognosis compared to the other subgroups (HR = 3.51; 95% CI: 2.74–4.49, *p* > 0.0001) (Table 2). A nomogram for clinical and research purposes was built using the coefficients by the multivariate model (Fig. 3).

## Discussion

We presented the results of a multicentric study aimed at building a prognostic model in melanoma patients with metastatic melanoma in the sentinel node by combining the information of six clinico-pathological variables including tumor burden (TB). Our findings show that TB significantly contributes to predict the prognosis of these patients.

To the best of our knowledge this is the largest series ever published with this aim, as previous studies have addressed the same issue using data from smaller series of patients. The negative prognostic value of TB, as maximum SN tumor size > 1 mm, was already assessed in terms of non-sentinel node positivity, disease free survival and melanoma specific survival by an international multicentric study of 1,539 SN positive melanoma patients [1].

Another study analyzing 104 SN positive patients who underwent CLND demonstrated that the 5-year melanoma specific survival for CLND-negative patients was 5 years as compared to 3.69 years in CLND positive patients. In this analysis, clinico-pathological parameters such as diameter of tumor deposit, distribution of metastatic focus within the sentinel node, ulceration and number of metastatic melanoma in the sentinel nodes were evaluated and the investigators found that TB > 4 mm and multifocal metastatic disease within the sentinel node were the most important variables that allowed an accurate prognostic stratification of patients [11]_._

On behalf of the EORTC-DeCOG, some authors developed prediction models for disease recurrence, distant metastasis (DM) and overall mortality analyzing a retrospective cohort of 1,080 patients. The resulting EORTC-DeCOG nomogram included parameters as ulceration, age, sentinel node TB, and Breslow: therefore, this study included only information deriving from the primary melanoma and the SNB, without considering the status of non-sentinel nodes [5].

In 2019, Satzger et al., assessing a total of 736 positive SN melanoma patients, demonstrated that TB, Breslow, ulceration and age are independent prognostic factors of melanoma specific survival. In detail, MSS was significantly better in patients with lower SN TB than in patients with higher SN TB (> 0.5 mm and > 1 mm) [17].

Overall, our results are consistent with the previous studies and confirm the prognostic value of TB. Our findings, along with the already existing literature on this subject, support the hypothesis that prognosis decays continuously with increasing maximum diameter of the larger metastatic deposit within the sentinel node: therefore, accumulating evidence strongly suggest that TB represents an important piece of information while stratifying the risk of these patients, even if the ideal cut-off needs to be determined.

In our opinion, CLND adds prognostic information as the status of non-sentinel nodes is an independent prognostic feature, as we have demonstrated not only in the present analysis but also previously [6, 11]. Therefore, CLND not only improves regional relapse-free survival (as demonstrated by the results of the MSLT-II) but also provides physicians with useful prognostic information [6–8].

For practical purposes, we generated a nomogram to easily personalize the prediction of patient prognosis. More precise risk stratification is important for adequate patient information on the severity of the disease and is especially useful for selecting patients who can benefit most from adjuvant therapy. Moreover, our nomogram could enable physicians to personalize the intensity of patients follow up as well as to optimize patient allocation within the frame of clinical trials.

Finally, we recognize that the present study has some limitations. First, it is a retrospective multicentric study with inherent bias: however the analyses were performed on complete cases without missing data. Second, we could not validate our results in an external series of patients, which would improve the assessment of model generalizability. Third, a good proportion of patients received interferon-alpha based adjuvant therapy, which could have potentially influenced the outcome, especially in patients with ulcerated melanoma. Moreover, some patients were treated with modern treatments (e.g., targeted therapy or immunotherapy) after disease recurrence, which could also have affected overall survival. We could not explore the impact of adjuvant therapy on OS because of insufficient data.

In addition, other potential prognostic factor as mitotic rate could not be incorporated in our analysis due to lack of complete data.

As regards the future perspective of survival predictive models, we believe that only the implementation of informative biomarkers will help improve the accuracy of current prognostic tools. Investigation on the molecular mechanisms underlying melanoma progression and aggressiveness has led to the identification of hundreds of potential such biomarkers [4–7, 18]; unfortunately, none of them has been so far associated with a predictive value independent of conventional clinico-pathological parameters. Therefore, more work is eagerly needed to make further advances in this field of investigation.

## Data Availability

The datasets generated and/or analysed during the current study are not publicly available due to ensuring the confidentiality and anonymity of the participants but are available from paolo.delfiore@iov.veneto.it on reasonable request.
